# The Adaptor Protein NumbL Is Involved in the Control of Glucolipotoxicity-Induced Pancreatic Beta Cell Apoptosis

**DOI:** 10.3390/ijms24043308

**Published:** 2023-02-07

**Authors:** Halesha D. Basavarajappa, Jose M. Irimia, Brandon M. Bauer, Patrick T. Fueger

**Affiliations:** 1 Department of Molecular and Cellular Endocrinology, Arthur Riggs Diabetes and Metabolism Research Institute, City of Hope, Duarte, CA 91010, USA; 2 Comprehensive Metabolic Phenotyping Core, Beckman Research Institute, City of Hope, 1500 E. Duarte Rd., Duarte, CA 91010, USA

**Keywords:** diabetes, epidermal growth factor, glucolipotoxicity, beta cell, apoptosis, Mig6, NumbL, NF-κB signaling, apoptosis, Notch signaling

## Abstract

Avoiding the loss of functional beta cell mass is critical for preventing or treating diabetes. Currently, the molecular mechanisms underlying beta cell death are partially understood, and there is a need to identify new targets for developing novel therapeutics to treat diabetes. Previously, our group established that Mig6, an inhibitor of EGF signaling, mediates beta cell death under diabetogenic conditions. The objective here was to clarify the mechanisms linking diabetogenic stimuli to beta cell death by investigating Mig6-interacting proteins. Using co-immunoprecipitation and mass spectrometry, we evaluated the binding partners of Mig6 under both normal glucose (NG) and glucolipotoxic (GLT) conditions in beta cells. We identified that Mig6 interacted dynamically with NumbL, whereas Mig6 associated with NumbL under NG, and this interaction was disrupted under GLT conditions. Further, we demonstrated that the siRNA-mediated suppression of NumbL expression in beta cells prevented apoptosis under GLT conditions by blocking the activation of NF-κB signaling. Using co-immunoprecipitation experiments, we observed that NumbL’s interactions with TRAF6, a key component of NFκB signaling, were increased under GLT conditions. The interactions among Mig6, NumbL, and TRAF6 were dynamic and context-dependent. We proposed a model wherein these interactions activated pro-apoptotic NF-κB signaling while blocking pro-survival EGF signaling under diabetogenic conditions, leading to beta cell apoptosis. These findings indicated that NumbL should be further investigated as a candidate anti-diabetic therapeutic target.

## 1. Introduction

Diabetes is a complex metabolic disorder affecting nearly 463 million people worldwide according to the International Diabetes Federation. The disease is characterized by increased blood glucose levels due to an imbalance in whole-body glucose homeostasis. Insulin-secreting pancreatic beta cells play a key role in controlling glucose homeostasis, and the depletion of functional beta cell mass is central to the manifestation of diabetes [1]. Hence, preventing beta cell loss or restoring functional beta cell mass remain major challenges in finding cures for diabetes.

Functional beta cell mass is modulated by multiple processes such as beta cell proliferation, beta cell hypertrophy, insulin secretory capacity, and apoptosis. During the development of type 2 diabetes (T2D), the pancreatic beta cell mass initially increases to maintain glucose homeostasis under chronic nutrient oversupply and peripheral insulin resistance conditions [2]. This increase in beta cell mass compensates for the increased insulin demand under excess glucose levels and is partially driven by low levels of ER stress through unfolded protein responses [3]. However, if the secretory burden persists, unresolved ER stress leads to beta cell death [4,5]. Moreover, the glucolipotoxic (GLT) milieu (elevated levels of both glucose and lipids), prevalent in diabetic conditions, further deteriorates the survival capacity of beta cells [6,7,8,9]. Once the functional beta cell mass declines, overt T2D arises [10]. Understanding the molecular events leading to pancreatic beta cell death under diabetogenic conditions is essential for discovering new therapies to prevent or cure diabetes. Apoptosis is a major pathway for the beta cell death [11] and is induced by an absence of pro-survival signals such as EGF signaling [12] and the activation of proapoptotic pathways such as NF-κB signaling [13].

It Is well established that GLT induces NF-κB signaling, and this pathway induces beta cell apoptosis [13,14]. Inactive NF-κB is bound to an inhibitor protein, IκB, and sequestered in the cytosol. The NF-κB pathway is activated when IκBα is phosphorylated and degraded to release free NF-κB. Once free of inhibition, NF-κB is phosphorylated and migrates to the nucleus to regulate transcription [15]. This degradation of IκBα is initiated by a sequence of events involving IKK, NEMO, and TRAF6 [15,16]. TRAF6, upon activation, is polyubiquitinated and then activates NEMO and IKK, which in turn phosphorylate IκBα [16]. However, how NF-κB signaling is activated under GLT is not clearly understood.

In addition to the induction of proapoptotic pathways, the absence of pro-survival signals also contributes to beta cell apoptosis. Among the many proliferative pathways characterized, epidermal growth factor receptor (EGFR) signaling is crucial for both beta cell proliferation and survival [12,17], and EGF treatment has been reported to induce beta cell proliferation and restore beta cell mass in rodents [18]. However, EGF signaling is impaired during beta cell stress, as occurs in T2D [19].

Previously, our group has demonstrated that Mig6, an inhibitor of EGFR signaling, plays a key role in beta cell apoptosis under diabetogenic conditions [19,20,21]. Upon induction, Mig6 binds to the EGF receptor and blocks pro-proliferative signaling [22]. Moreover, we have observed that under GLT conditions, Mig6 levels are elevated, and EGFR signaling is inhibited (Y-C Chen and PT Fueger, personal communications). Although Mig6 is considered to be a molecular brake for proliferation, it also antagonizes beta cell survival and impairs beta cell function [19,20]. Thus, strategies for suppressing Mig6 action in beta cells could improve the retention of functional beta cell mass.

The objective of this study was to better understand how GLT regulates beta cell death by investigating Mig6 binding partners. We discovered that Mig6 dynamically interacted with the adaptor protein NumbL, a component of Notch signaling [23,24]. Furthermore, we delineated a novel role for NumbL in beta cell apoptosis, and we propose that reducing NumbL could be exploited to prevent beta cell death under glucolipotoxic conditions.

## 2. Results

### 2.1. Mig6 Dynamically Interacts with NumbL Protein

**Figure 1:**
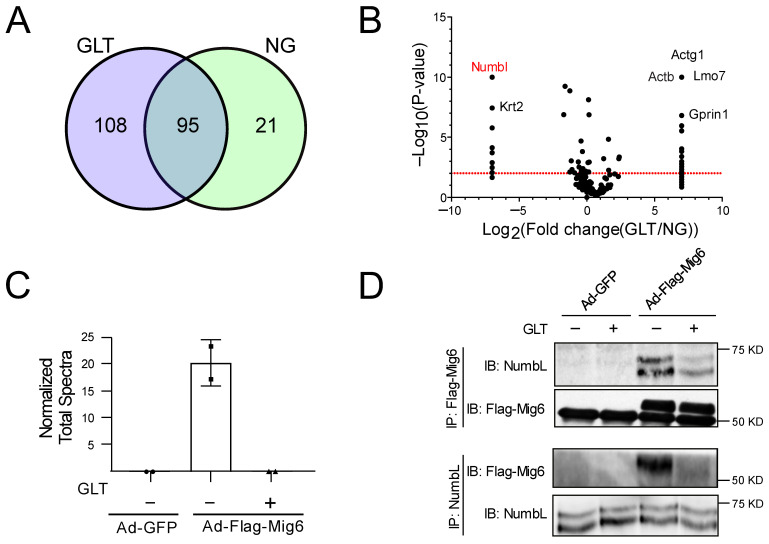
Mig6 interacted with NumbL under NG conditions, and this interaction was disrupted under GLT conditions. (**A**) Venn diagram depicting number of proteins pulled down with Flag-Mig6 under NG and GLT conditions. (**B**) The fold change in number of peptide counts of each pulled down protein under GLT and NG conditions is plotted against *p*-values of Fisher’s exact *t*-test to screen for proteins with significantly altered binding patterns with Mig6. (**C**) The number of unique peptide counts of NumbL protein identified from eluate samples (normalized to total spectral count of the eluate samples). (**D**) Representative immunoblot from three separate co-immunoprecipitation experiments confirming the interaction between NumbL and Mig6 under NG and GLT conditions. GLT, glucolipotoxicity; NG, normal glucose. All the mass spectrometry and immunoblot images are representative from at least two independent experiments.

Although Mig6 was initially considered as a stress-inducible gene, increasing reports indicate that it contributes to beta cell death under stressed conditions [19,20]. To clarify the mechanisms underlying diabetogenic beta cell death, we sought to identify Mig6-associating partners in a GLT cell culture model, which mimics the diabetic milieu [6,25]. To identify the potential interacting protein partners of Mig6, we overexpressed FLAG-tagged Mig6 protein in Ins-1-derived 832/13 cells using adenovirus and exposed the cells to NG or GLT conditions for 12 h. Using an anti-FLAG antibody, we immunoprecipitated Mig6 along with its interacting proteins and identified the associating proteins using mass spectrometry (Figure 1A). As Mig6 is an adaptor protein, it was expected that multiple proteins would bind to Mig6 (Figure 1A). Hence, we screened for interacting proteins whose binding pattern with Mig6 was significantly altered with changes in the glucolipotoxic milieu of the culture conditions (Figure 1B). Additionally, protein components of the cytoskeleton system such as actin, spectrin, and myosin would be ignored. Given the goals of the screen, we focused on the proteins reported to be involved in EGF and/or NF-κB signaling. We then confirmed the results by immunoblot analysis and reverse co-immunoprecipitation experiments (Figure 1C,D). We observed that Mig6 interacted dynamically with NumbL protein, showing a strong association under NG conditions and a weaker association under GLT conditions (Figure 1C,D). Specifically, when the complexes from AdCMV-Flag-Mig6-treated cells cultured in control and GLT conditions were immunoprecipitated with anti-Flag and blotted with anti-NumbL, the GLT conditions decreased NumbL expression (1.00 ± 0.40 vs. 0.55 ± 0.27 relative units, *p* < 0.05). Similarly, when the complexes from the AdCMV-Flag-Mig6-treated cells cultured in control and GLT conditions were immunoprecipitated with anti-NumbL and blotted with anti-Flag to detect endogenously expressed Mig6, the GLT conditions decreased Mig6 expression (1.00 ± 0.18 vs. 0.46 ± 0.11 relative units, *p* < 0.05).

**Figure 2:**
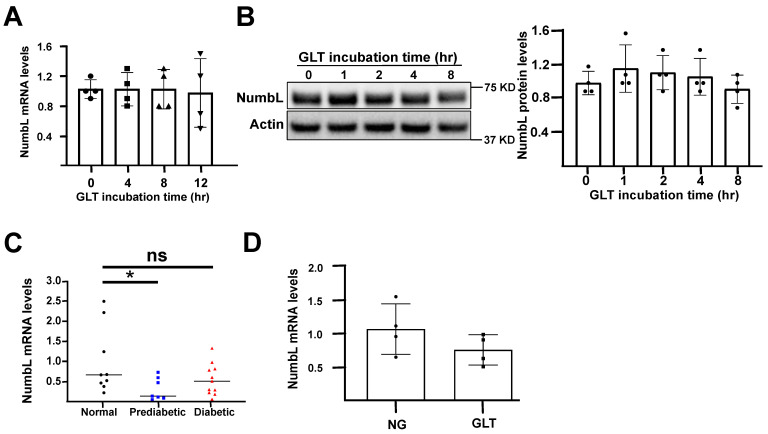
NumbL expression levels were unchanged in beta cells under GLT conditions. (**A**) mRNA levels of NumbL in 832/13 cells after culturing in GLT conditions for the indicated time periods. (**B**) Protein levels of NumbL in 832/13 cells after culturing in GLT conditions for indicated time periods. (**C**) mRNA levels of NumbL in human islets obtained from normal, prediabetic, and diabetic patients. * *p* < 0.05 vs. normal; ns = not significant. (**D**) mRNA levels of NumbL in human islets after culturing in NG and GLT conditions for 48 h. All data are represented as mean +/− standard deviation and were compared by ANOVA. The data are representative from at least three independent experiments.

To verify that the decreased binding was not due to the decreased expression levels of NumbL, we measured both the mRNA and protein levels of NumbL under both NG and GLT conditions at various time points (Figure 2). We detected no change in the levels of NumbL in the 832/13 cells exposed to GLT conditions, suggesting that the interaction between Mig6 and NumbL was dynamic and not determined solely by the protein abundance. Similarly, the primary human islets cultured in GLT conditions for 48 h exhibited no change in NumbL expression levels (Figure 2D). Further, we measured the NumbL mRNA levels in primary islets from patients with pre-diabetes (HbA1c level between 5.7–6.4%) and T2D (HbA1C levels > 6.4%) (Figure 2C). Although there were no significant changes in the expression levels between the controls (HbA1C levels < 5.7%) and patients with diabetes, there was a surprisingly significant decrease in NumbL between the control and pre-diabetic groups. Further investigation is required to understand this difference in the NumbL levels between the control and pre-diabetic subjects.

### 2.2. Downregulation of NumbL Prevents Glucolipotoxicity-Induced Beta Cell Apoptosis

**Figure 3:**
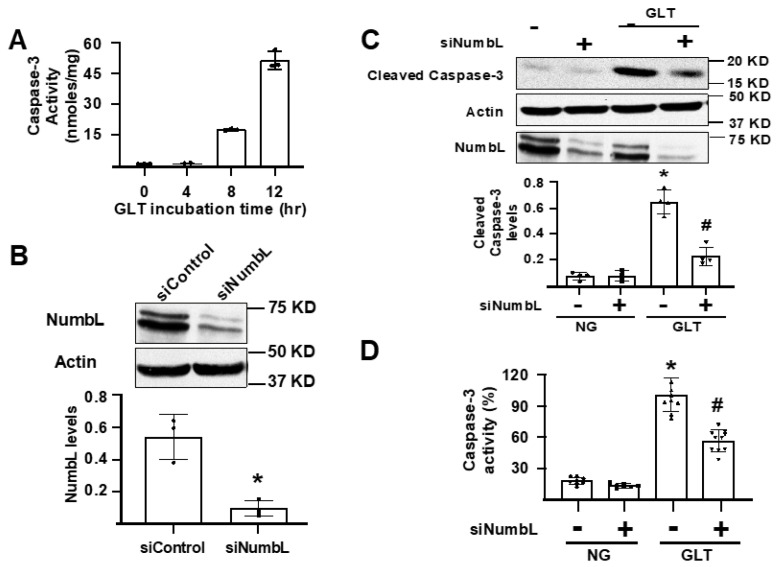
siRNA-mediated knockdown of NumbL inhibited GLT-induced apoptosis of Ins1 832/13 cells. (**A**) Cleaved caspase-3 activity after exposure of cells to GLT for the indicated time periods. (**B**) Representative blot and quantification of NumbL protein levels from cells treated with a control RNAi (siControl) or RNAi to NumbL (siNumbL). * *p* < 0.05 (*t*-test). (**C**) Representative blot and quantification of cleaved caspase-3 protein from cells treated with siNumbL either under NG or GLT for 12 h. * *p* < 0.05 vs. siControl treated under NG; # *p* < 0.05 vs. siControl treated under GLT (ANOVA). (**D**) Cleaved caspase-3 activity in siRNA-treated cells after culturing in NG and GLT conditions for 12 h. * *p* < 0.05 vs. siControl treated with NG; # *p* < 0.05 vs. siControl treated with GLT. The data are representative from at least three independent experiments.

As Mig6 has been implicated in inducing beta cell apoptosis under stressed conditions, we investigated the role of its interacting protein, NumbL, in beta cell apoptosis under GLT conditions. As depicted in Figure 3A, the GLT conditions induced apoptosis in the 832/13 cells in a time-dependent manner, as measured by the cleaved caspase-3 activity. To test the role of NumbL, we used siRNA knockdown and confirmed an over 80% suppression of NumbL expression by immunoblot analysis (Figure 3B). The activation of apoptosis with GLT was significantly reduced by the siRNA-mediated suppression of NumbL, as measured by the levels of cleaved caspase-3 (Figure 3C) and cleaved caspase-3 activity (Figure 3D). 

The prevention of beta cell apoptosis under GLT prompted us to test the extent to which NumbL downregulation resolved ER stress induced by GLT. However, there was no significant change in the GLT-induced ER stress between the siRNA control and siNumbL groups, as measured by the levels of phospho-eIF2a (Figure S1).

### 2.3. Reducing NumbL Does Not Enhance Beta Cell Proliferation and Insulin Secretion

In lung cancer cell lines, the suppression of NumbL has been reported to promote cell proliferation [26]. Hence, we sought to determine the extent to which NumbL knockdown in 832/13 cells induces beta cell proliferation using an EdU incorporation assay. However, NumbL knockdown did not induce significant proliferation as compared to the control siRNA-treated cells under either NG or GLT conditions (Figure S2A). This result was further corroborated by the observation that the knockdown of NumbL did not rescue the glucolipotoxicity-induced impairment of EGF signaling, a key pro-proliferative pathway in beta cells (Figure 2B). However, it is interesting to note that NumbL knockdown significantly increased EGFR phosphorylation under NG conditions. Further, NumbL depletion did not affect beta cell function, as measured by glucose-stimulated insulin secretion (Figure S2C).

### 2.4. NumbL Reduction Does Not Activate Notch Signaling in 832/13 Cells

NumbL, along with its homologous protein Numb, were originally characterized as Notch signaling inhibitors and cell-fate-determining proteins [23,24,27]. Hence, we tested whether the knockdown of NumbL activates Notch signaling in response to GLT conditions by monitoring the levels of Hes-1, a downstream target gene of Notch signaling. Whereas Hes-1 expression was increased under GLT conditions in the 832/13 cells, the knock-down of NumbL did not affect Notch signaling (Figure S3A). Interestingly, we noticed that only the siRNA directed against Numb and not NumbL increased the Hes-1 expression levels under GLT conditions. These observations indicated that, under the studied conditions, either NumbL may have not participated in Notch signaling in the 832/13 cells or that Numb was the major mediator of Notch inhibition in the 832/13 cells. These observations contrasted with the functions of NumbL observed in other cell types [24], indicating tissue-specific variations in the functions of NumbL.

### 2.5. Downregulation of NumbL Prevents Glucolipotoxicity-Induced Activation of the NF-κB Pathway

Several groups have reported that NF-κB pathway activation leads to apoptosis in beta cells under diabetogenic conditions [13,28,29]. In its inactive state, NF-κB is bound to the IκB inhibitor protein and is sequestered in the cytosol. Upon activation, IκBα is phosphorylated and degraded by the proteasome. Once free of inhibition, NF-κB is phosphorylated and migrates to the nucleus where it exerts its transcriptional function.

**Figure 4:**
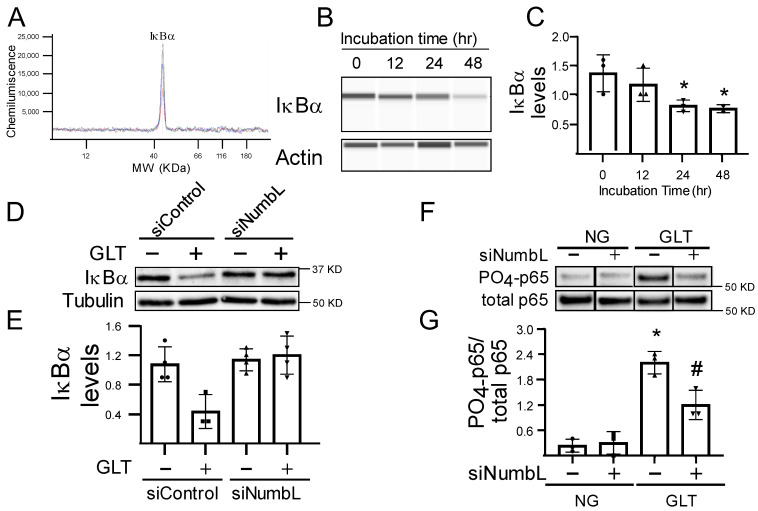
NumbL downregulation prevented GLT-mediated activation of NF-κB signaling. (**A**,**B**) Representative histogram and immunoblot of IkBa protein using the capillary Western blot system from human islets treated with GLT conditions for the indicated time periods. (**C**) Quantification of IkBa protein. The quantification data are from three independent experiments. * *p* < 0.05 vs. ‘0 h GLT’ group. (**D**,**E**) Representative immunoblot and quantification of IkBa protein from siRNA-treated 832/13 Ins1 cells cultured in NG or GLT conditions for 12 h. * *p* < 0.05 vs. siControl group under NG. (**F**,**G**) Representative immunoblot and quantification of phospho-p65 protein from siRNA-treated 832/13 Ins1 cells cultured in NG or GLT conditions for 12 h. * *p* < 0.05 vs. siControl group under NG; # *p* < 0.05 vs. siControl group under GLT. The data are representative from at least three independent experiments.

When either the human islets (Figure 4A–C) or the 832/13 cells (Figure 4D–G) were incubated in GLT conditions, we observed a significant activation of the NF-κB pathway, as measured by the decreasing levels of IκB and the increased phosphorylation of p65, which is activated in the NF-κB pathway. However, the depletion of NumbL in beta cells (Figure 4D–G) significantly prevented the degradation of IκBα and the phosphorylation of p65, indicating that the inactivation of NumbL in beta cells could increase the resistance to GLT-induced NF-κB pathway activation. These data confirmed that NumbL mediated GLT-induced NF-κB pathway activation. 

### 2.6. NumbL Interacts with TRAF6 in a Context-Dependent Manner

**Figure 5:**
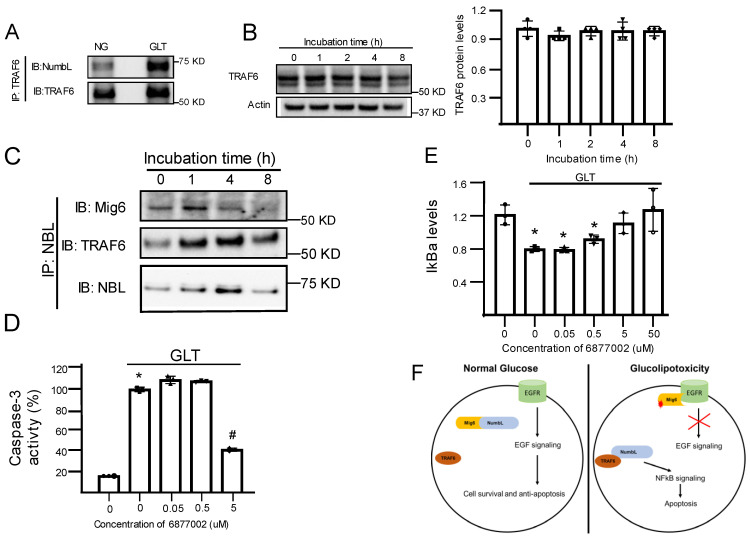
NumbL interaction with TRAF6 was increased under GLT conditions. (**A**) Representative blot of TRAF6 proteins pulled down using an anti-NumbL antibody. (**B**) Representative blot of TRAF6 protein and quantification of TRAF6 protein levels in cells treated with GLT for the indicated time periods. (**C**) Representative immunoblot from co-immunoprecipitation experiments confirming the interaction between NumbL, TRAF6, and Mig6 under GLT conditions. (**D**,**E**) Effect of TRAF6 inhibitor 6877002 on the levels of cleaved caspase-3 and IkBa proteins in beta cells exposed to GLT for 12 h. (**F**) Proposed model of interactions between Mig6, NumbL, and TRAF6 on EGFR and NFkB signaling in beta cells. * *p* < 0.05 vs. DMSO control under NG conditions and # *p* < 0.05 vs. DMSO control under GLT conditions. The data are representative from at least three independent experiments.

As NumbL downregulation inhibits NF-κB signaling under GLT, we evaluated the mechanism through which NumbL is linked to NF-κB. Several groups have reported that NumbL interacts with TRAF6 and modulates NF-κB signaling [30,31]. Thus, we tested the dynamics of the interactions between NumbL and TRAF6 under GLT conditions in beta cells. As reported in Figure 5A, in the beta cells, endogenous NumbL and TRAF6 interacted under NG conditions, and this interaction was increased nearly two-fold under GLT conditions. To determine if this increased binding was simply due to the increased expression of TRAF6, we measured TRAF6 protein levels under GLT conditions over different time points (Figure 5B) and noted no significant increase in the TRAF6 expression levels, indicating an increased interaction between NumbL and TRAF6 under GLT conditions. Meanwhile, we observed a time-dependent decrease in the binding of NumbL and Mig6, while the binding between NumbL and TRAF6 was increased under GLT conditions (Figure 5C). In order to further test the role of TRAF6 in the activation of NF-κB signaling and apoptosis under GLT, we treated beta cells under GLT conditions with different concentrations of compound 6877002, an inhibitor of TRAF6 activation [32], and monitored the levels of cleaved caspase-3 (Figure 5D) and IkBa levels (Figure 5E). We observed that compound 6,877,002 decreased both NF-κB activation and beta cell apoptosis in a dose-dependent manner.

## 3. Discussion

In the present study, we discovered that Mig6 binded to NumbL under NG conditions, and this interaction was disrupted under GLT conditions. Conversely, NumbL interacted more abundantly with TRAF6 under GLT conditions than NG conditions. In addition, we established that NumbL downregulation prevented both the activation of NF-κB signaling and beta cell apoptosis under GLT condition. The combined interactions of Mig6, NumbL, and TRAF6, along with the pro-apoptotic nature of NumbL, may indicate that these proteins regulate beta cell apoptosis under GLT conditions. Further, we propose a model that, under NG conditions, Mig6 is locked in an inactive complex with NumbL and thereby pro-survival EGF signaling is kept active. When beta cells are exposed to GLT conditions, this Mig6-NumbL complex is disrupted to release Mig6 and NumbL. The released Mig6 protein then binds to EGFR and inhibits EGF signaling. Meanwhile NumbL binds to and activates TRAF6 protein to induce NF-κB signaling (Figure 5E). The results presented here were consistent with a model wherein GLT caused NumbL to switch from a Mig6-containing complex to a TRAF6-activating complex, thereby increasing pro-apoptotic signaling through NF-κB in beta cells (Figure 5E).

Given the known role for EGF signaling in pancreatic beta cell survival and proliferation [12,17] and the ability of Mig6 to inhibit this pathway [33], we hypothesized that the Mig6-interacting partner NumbL would regulate EGF signaling. To our surprise, NumbL depletion significantly increased EGF signaling under NG conditions and did not alter EGF signaling under GLT conditions (Figure S2B). This increased EGF signaling under NG conditions can be partially explained by the fact that NumbL, along with Numb protein, also participates in Erbb receptor (EGFR belongs to the Erbb family of receptors) degradation, as reported by others [27]. The observation that EGF signaling was increased under NG conditions and impaired in GLT-treated cells even after NumbL depletion indicated that the free Mig6 did not directly inhibit EGFR after its release from the Mig6–NumbL complex. We believe that the free Mig6 protein in NumbL-depleted cells still needs to undergo posttranslational modifications before binding to EGFR and exerting its inhibitory function. Our group observed that, under GLT conditions, Mig6 was upregulated and was responsible for EGFR signal impairment (unpublished data). Consistent with this idea, other groups have reported that Mig6 undergoes extensive phosphorylation events before inhibiting EGF signaling [33]. Hence, further investigations are required to decipher the role of NumbL in EGF signaling in beta cells. It remains to be determined whether other factors, such as the post-translational modification of Mig6, play a role in mediating the dynamic relationship between Mig6 and NumbL under GLT conditions. It also remains to be determined if the NumbL–Mig6 complex locks Mig6 in an inactive form and whether other pathways are involved in GLT-induced beta cell apoptosis. Bagnati et al. reported that under GLT conditions, there is increased expression of CD40 receptors in beta cells [13], and CD40-TRAF6 interactions are known to activate the NF-κB pathway [13,34]. The accumulation of the NumbL–TRAF6 complex and the initiation of NF-κB signaling under GLT conditions suggests that NumbL plays a key role in activating the NF-κB pathway. However, further investigations are warranted to understand how the TRAF6–NumbL complex activates the NF-κB pathway. We hypothesize that NumbL promotes the polyubiquitination of TRAF6, which in turn activates the IKK complex. It also remains to be determined if the CD40 receptor binding to this NumbL–TRAF6 complex is required for NF-κB activation.

TRAF6 activity depends on its polyubiquitination [16], and it is not clear if TRAF6, being a ubiquitin ligase, ubiquitinates itself or if other factors are involved [15,16,30]. Swarnker et al. determined that NumbL promotes the polyubiquitination of TRAF6 and NEMO in osteoclasts [30]. As polyubiquitinated TRAF6 is required for the activation of NF-kB signaling and NumbL promotes the polyubiquitination of TRAF6, it is intuitive to predict that under GLT, NumbL activates TRAF6 to induce NF-kB, thereby causing beta cell apoptosis. However, Swarnker et al. reported that NumbL negatively regulates NF-κB signaling in osteoclasts, whereas we observed NumbL activating NF-κB signaling. The contradictions in these results can be explained partly by the difference in the context of the study, the cell type, and the incubation time of the experiments. In our study, we treated the beta cells with GLT for a short period of time (1 to 12 h), whereas Swarnker et al. used a longer-duration cell model (4 d) to study the effect of NumbL on TRAF6. In beta cells, NF-κB activation starts within a couple of hours after exposure to GLT, and apoptosis is induced after approximately 8 h of exposure to GLT. Because polyubiquitinated TRAF6 first initiates the NF-κB pathway and then undergoes degradation, it is possible that we captured the initial phase of the events, such as TRAF6 ubiquitination, IkBa degradation, and NF-κB activation, which might lead to the apoptosis of beta cells. Further investigations are required to delineate these molecular events.

Despite revealing new aspects of beta cell death in response to GLT, there were a few limitations worth discussing. First, much of the work presented here was from a well-characterized rodent beta cell line, and it may not directly translate to rodent and human primary islets. It would be an important next step to validate the results (e.g., the interaction of NumbL and TRAF6) in human tissues. Second, it is unknown what the specific molecular mechanisms are that drive the dissociation of the adaptor proteins NumbL and Mig6 during GLT exposure. Mig6 is known to undergo numerous post-translational modifications, including serine/threonine or tyrosine phosphorylation at numerous residues as well as ubiquitination [35,36,37]; similarly, NumbL undergoes phosphorylation at Ser304 [38]. Given that NumbL and Mig6 can serve as molecular scaffolds in signaling cascades, these post-translational modifications as well as their interactions with other binding partners can dictate the specific proteins forming a complex.

Overall, NumbL depletion was beneficial for the survival of beta cells under diabetogenic conditions. To support this idea, we also observed that NumbL was significantly decreased in human islets from prediabetic subjects, where there was an expansion of the functional beta cell mass. However, more investigations are warranted to validate NumbL as a therapeutic target to treat diabetes.

## 4. Materials and Methods

### 4.1. Materials

The following antibodies were purchased from Cell Signaling Technologies (Denver, CO, USA): phospho-EGFR, EGFR, p65, phospho-p65, phospho-eIF2a, eIF2a, caspase-3, and cleaved caspase-3 (Table S1). Antibodies to NumbL and TRAF6 were obtained from SantaCruz Biotechnology (Dallas, TX, USA). The sequence of siRNAs (mission siRNAs purchased from Sigma-Aldrich, St. Louis, MO, USA) used against NumbL was 5′-GAACUCACCUUUCAAACGU[dT][dT]-3′] and 5′-ACGUUUGAAAGGUGAGUUC[dT][dT]-3′, and that of Numb was 5′-GAAGACUGAUUUCCCAAUA[dT][dT]-3′ and 5′-UAUUGGGAAAUCAGUCUUC[dT][dT]-3′; MISSION siRNA Universal Negative Control was used as a control. Taqman probes for NumbL, Numb, and Hes-1 genes, cell culture media, RPMI-1640, and CMRL-1066 were obtained from Thermo Fisher Scientific (Waltham, MA, USA). All the other reagents, if not indicated, were purchased from Sigma-Aldrich (St. Louis, MO, USA).

### 4.2. Human Islet Cell Culture

Cadaveric human islets were procured from the Southern California Islet Resource Center (City of Hope, Duarte, CA, USA) and cultured for 24 h in CMRL-1066 medium supplemented with 10% fetal bovine serum (FBS), 50 U/mL penicillin, and 50 μg/mL streptomycin before starting the GLT experiments. Then, the human islets were cultured in CMRL-1066 medium containing either 5 mM glucose (NG conditions) or 25 mM glucose and 0.4 mM sodium palmitate (GLT conditions) for various time points, as indicated in the figure legends. The islets were collected at the indicated time points and washed twice in PBS before cell lysis in RIPA buffer for immunoblot experiments. The protein lysates were loaded in a Wes instrument (ProteinSimple, Santa Clara, CA, USA) according to the manufacturer’s protocol.

### 4.3. 832/13 Cell Culture and Transfection

Ins-1-derived 832/13 cells were cultured as described previously in Ins-1 medium consisting of RPMI-1640 medium supplemented with 10% FBS, 50 U/mL penicillin, 50 µg/mL streptomycin, 10 mM HEPES pH 8.0 buffer, 2 mM L-glutamine, 1 mM sodium pyruvate, and 0.05 mM 2-mercaptoethanol [39]. The cells were seeded in 6-well tissue culture plates and, after overnight culturing, were treated with 50 nM of either Mission control Negative siRNA (siControl) or siRNAs specific against Numb or NumbL (siNumbL) for 48 h. Then, the medium was replaced with either normal glucose (NG) or GLT media for the indicated time periods, as described in the figure legends.

### 4.4. Glucolipotoxicity Experiments with 832/13 Cells

832/13 cells were grown in Ins-1 medium until confluent and then cultured in Ins-1 medium containing 25 mM glucose and 0.4 mM palmitate for various time periods, as indicated in the figure legends. For NG conditions, the cells were incubated in Ins-1 medium containing 11 mM glucose.

### 4.5. Co-Immunoprecipitation of Mig6 Binding Partners

832/13 cells were grown in 15 cm round tissue culture dishes to near confluence and then treated with adenoviruses expressing FLAG-tagged rat Mig6 (Ad-Flag-Mig6) or GFP (Ad-GFP) for 48 h. The cells were then treated either 5 mM glucose or 25 mM glucose and 0.4 mM palmitate for 12 h. After the incubation, the cells were collected and sonicated for 10 s with 60% amplitude in cell lysis buffer (20 mM Tris PH 7.2, 150 mM NaCl, 1% NP-40, 1 mM EDTA, 1 mM EGTA, 1 mM NaF, 1 mM sodium orthovanadate, 1 mM PMSF, and Roche’s protease inhibitor tablet (Sigma-Aldrich, St. Louis, MO, USA, catalog # 4693159001)). The cell lysate was centrifuged at 14,000× *g* for 10 min to collect the supernatant. Protein A/G sepharose beads were equilibrated with cell lysis buffer and incubated with the supernatant for 12 h at 4 °C with rotation. After the incubation, the beads were collected by centrifugation at 1000× *g* for 5 min and extensively washed in cell lysis buffer to remove non-specific binding proteins. The beads were then incubated with elution buffer (cell lysis buffer + 500 μg/mL FLAG peptide) to specifically elute FLAG-tagged Mig6 and its binding partners. The eluate was then further processed, and peptides were identified with mass spectrometric analysis by the Multi-Omics Mass Spectrometry Core at the City of Hope.

### 4.6. Apoptosis Assay (Cleaved Caspase-3 Activity Assay)

832/13 cells were treated with either NG or GLT for the indicated time points. After the incubation, the cells were lysed in M-PER mammalian protein extraction reagent without protease inhibitors, and the supernatant was collected after centrifugation at 5000× *g* for 15 min. The protein concentration of the supernatant was determined by the BCA method. In a 96-well black microplate, 50 μg of protein was incubated with 25 μM of caspase-3 fluorometric substrate (Ac-Asp-Glu-Val-Asp-AMC), and the plate was read at 37 °C for 1 h with excitation and emission wavelengths of 360 nm and 460 nm, respectively. In the same plate, a standard curve was prepared with AMC (7-Amino-4-methylcoumarin) to quantify the caspase-3 activity.

### 4.7. Proliferation Assay (EdU Incorporation)

Cell proliferation was quantified using an EdU incorporation assay, as described before [40,41]. Briefly, nearly 5000 cells were seeded in 8-well chamber plates and treated with either siControl or siNumbL for 48 h and then incubated in GLT conditions for 12 h in the presence of 10 μM EdU. The cells were then fixed with 4% formaldehyde for 30 min and permeabilized with 0.5% Triton X-100 for 15 min. The cells were then incubated for 30 min with detection reagent (100 mM Tris-Cl pH 8.5, 0.5 mM CuSO_4_, 25 μM sulfo-Cyanine 3 azide, and 50 mM ascorbic acid). After the incubation, the cells were washed in PBS and mounted using Vectashield mountant containing DAPI dye. Cell images were captured using a fluorescence microscope, and the number of DAPI- and EdU-stained cells was counted using the ImageJ software (Bethesda, MD, USA).

### 4.8. Immunoblotting

After treatment, the cells were lysed in RIPA buffer and centrifuged at 5000× *g* for 20 min to collect the supernatant. Protein concentrations were determined by the BCA method, and equal amounts of protein were run on SDS-PAGE gels. The proteins were then transferred to PVDF membranes and immunoblotted. The following primary antibodies with a dilution factor of 1:1000 were used for immunoblotting: caspase-3, NumbL, Numb, p65, phospho-p65, IκBα, FLAG, TRAF6, γH2AX, phospho-eIF2a, and total eIF2a. Tubulin and actin antibodies were used at 1:5000 dilutions. The following secondary antibodies were used: goat anti-mouse IgG-HRP conjugate (1:5000) and goat anti-rabbit IgG-HRP conjugate. The blots were developed using Amersham ECL detection reagent (GE Healthcare, Chicago, IL, USA). For the samples derived from human islets, with a more limited amount of protein available, we performed the immune assay using the capillary Western blot system (Wes, Protein Simple; San Jose, CA, USA) due to its sensitivity compared to the traditional Western blot method. All experimental steps were carried out according to the manufacturer’s instructions.

### 4.9. Glucose-Stimulated Insulin Secretion (GSIS) Assay

832/13 cells were treated with siControl or siNumbL as described above (Section 2.3), and 48 h after treatment, a GSIS assay was performed as previously described [42,43]. Briefly, the cells were incubated in low-glucose (2.5 mM) KRBB buffer for 1 h and then incubated in KRBB buffer containing low glucose (2.5 mM) or high glucose (15 mM) amounts for 1 h. The cells and media were separated after the incubation and processed for quantification of the insulin levels using an RI-13K Rat Insulin RIA Kit (Millipore) according to the manufacturer’s protocol.

### 4.10. Gene Expression Analysis

Gene expression was measured using quantitative real-time PCR. RNA from treated Ins1 832/13 cells and human islets were isolated using TRIzol reagent (Invitrogen) and a RNeasy Micro kit (Qiagen), respectively, according to manufacturer’s instructions. cDNA was synthesized using random primers and iScript reverse transcriptase. qPCR was performed in a 20 uL volume in a 96-well plate with Fast Advanced Master Mix and TaqMan probes on a ViiA7 thermal cycler (Life Technologies). Primer/probe sets used were as follows: rat NumbL (Rn01476538_m1), human NumbL (Hs00191080_m1), human Numb (Hs01105433_m1), rat Numb (Rn01518088_m1), rat Hes-1 (Rn01404555_m1), and housekeeping controls GAPDH and actin. The data were analyzed using the ΔΔCt method. The expression levels of genes were normalized to the housekeeping genes.

### 4.11. Statistical Analysis

All the quantified data are presented as means ± SEM. Student’s t-tests for analysis of two groups and ANOVA for analysis of more than two groups were employed to detect differences between groups. Tukey’s post hoc test was conducted after the ANOVA analyses, and *p*-values of < 0.05 were considered statistically significant.

## 5. Conclusions

The results of the present study suggested that NumbL and Mig6 were in an inactive complex under NG conditions, and under GLT conditions, they separated, and Mig6 decreased EGF signaling while NumbL increased NF-κB signaling. In addition, we uncovered a novel role of NumbL in beta cell apoptosis under GLT conditions. Future investigations will evaluate NumbL as a potential therapeutic target to prevent beta cell death under diabetogenic conditions.

## Data Availability

Data will be made available upon request.

## Supplementary Materials

The following supporting information can be downloaded at: https://www.mdpi.com/article/10.3390/ijms24043308/s1.
