# Supplementary material for: The Adaptor Protein NumbL Is Involved in the Control of Glucolipotoxicity-Induced Pancreatic Beta Cell Apoptosis
Source: Int J Mol Sci. 2023 Feb 7;24(4):3308. doi: 10.3390/ijms24043308 (PMC9959170; doi:10.3390/ijms24043308)
Supplement: Supplementary file 1 [file ijms-24-03308-s001.zip › ijms-2172555-supplementary.pdf]

Supplementary Files:

Table S1. The details of the antibodies used in the manuscript:.

Antibody	Company	Catalog #	Dilution
Phospho-EGFR (Y1173)	Invitrogen	44794G	1:1000
EGFR	Cell Signaling Technology	2232	1:1000
NUMB	Cell Signaling Technology	2756	1:1000
NUMBL	Santa Cruz Biotechnology	SC-390590	1:1000
Phospho-p65	Cell Signaling Technology	3031	1:1000
p65	Cell Signaling Technology	8242	1:1000
Phospho-eIF2a (S51)	Cell Signaling Technology	9721	1:1000
eIF2a	Cell Signaling Technology	5324	1:1000
Caspase-3	Cell Signaling Technology	9662	1:1000
Cleaved Caspase-3	Cell Signaling Technology	9664	1:1000
TRAF6	Santa Cruz Biotechnology	SC-8409	1:1000
Tubulin	Cell Signaling Technology	2144	1:1000
Actin	Millipore Sigma	MAB1501	1:1000

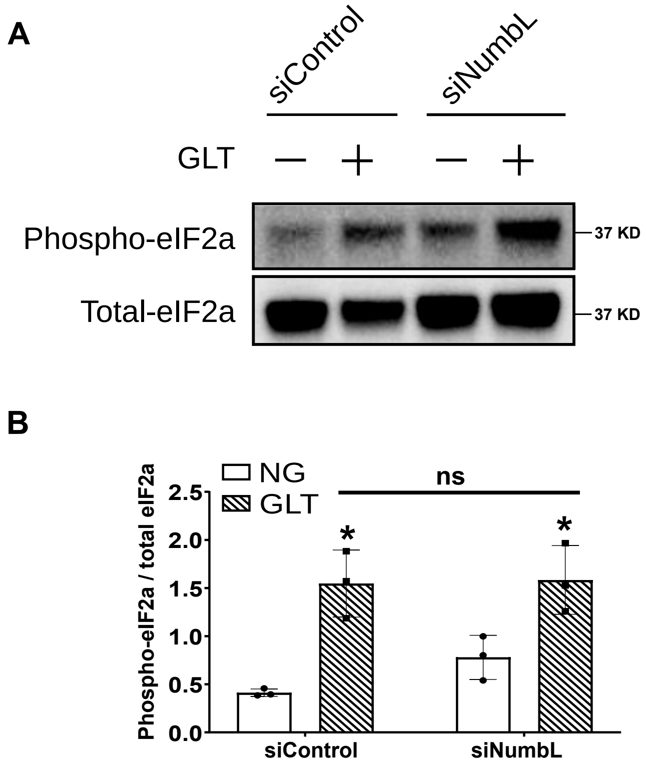

Figure S1: siRNA-mediated knock down of NumbL does not prevent GLT-induced ER-stress. (A) & (B) representative immunoblot and quantification of ER-stress marker proteins phospho-eIF2α and total eIF2α. 832/13 Ins1 cells were treated with NG or GLT conditions for 12 hours. *p<0.05 vs. siControl group under NG & ns; p>0.05. The data are representative from at least three independent experiments.

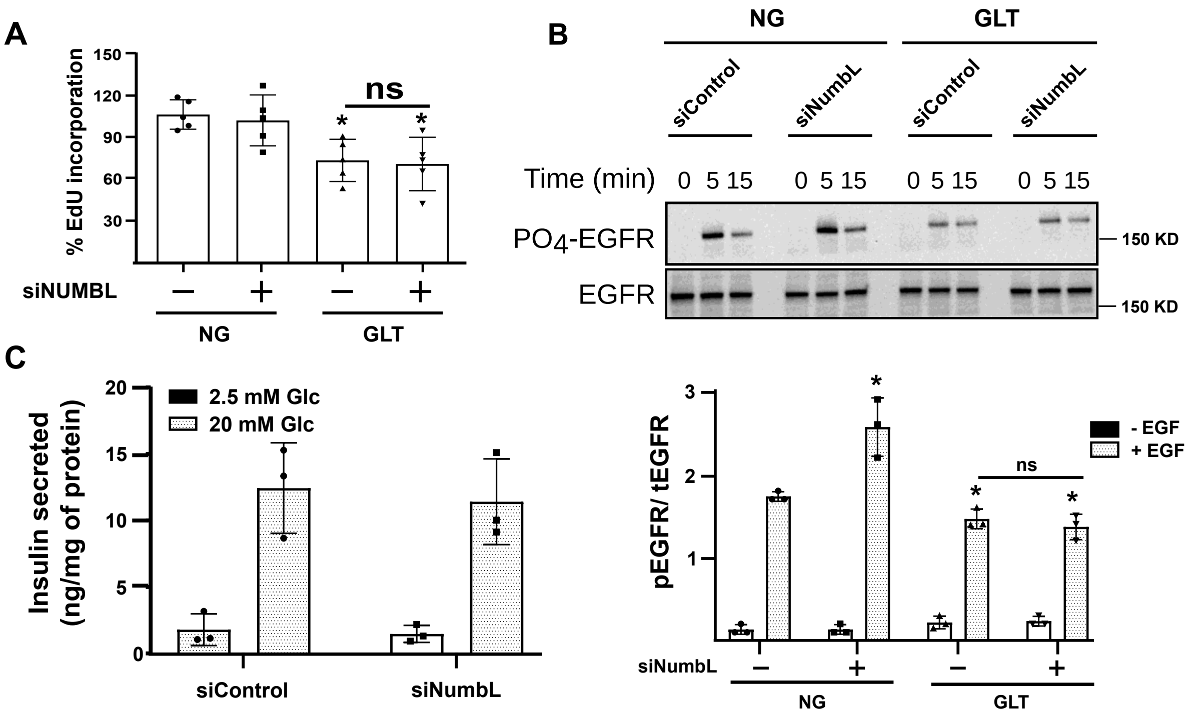

Figure S2. NumbL down regulation does not affect the proliferation and insulin secretion capacity of beta cells. (A) Quantification of beta cell proliferation using EdU incorporation assay in siNumbL- or siControl-treated cells under NG or GLT conditions for 12 h. (B) NumbL down regulation does not rescue impairment of EGF signaling under GLT. Representative immunoblot of phospho-EGFR and total EGFR proteins from siNumbL- or siControl-treated cells cultured under NG or GLT for 12 h. After incubation, the cells were exposed to either 0, 5 or 15 min of EGF ligand. Quantification of phospho-EGFR levels from EGF treated samples for 0 (-) and 5 min (+) are shown. *p<0.05 vs. siControl with 5 min EGF group under NG. (C) Quantification of glucose-stimulated Insulin secretion capacity of Ins1 832/13 cells in siNumbL- or siControl-treated cells. *p<0.05 vs. siControl group under NG & ns; p>0.05. The data are representative from at least three independent experiments.

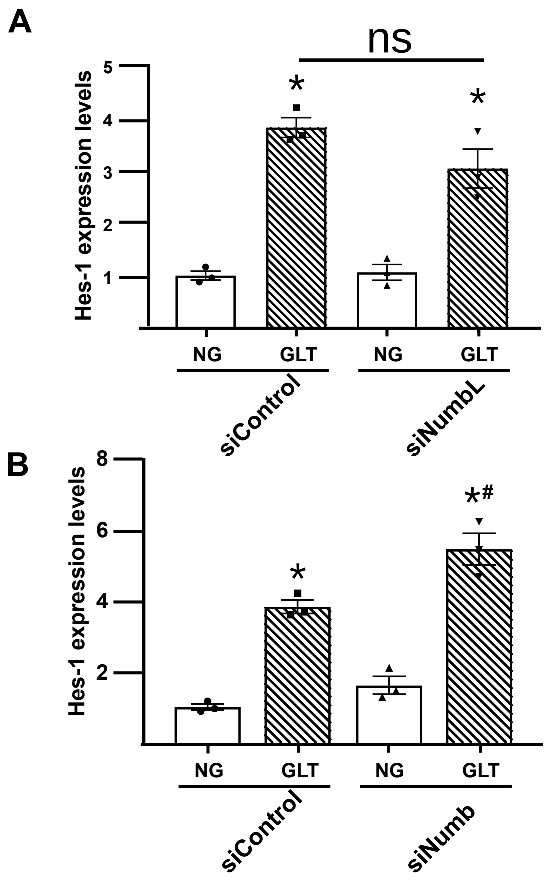

Figure S3: NumbL down regulation does not affect Notch signaling in beta cells. (A) Quantification of Hes-1 mRNA levels in siNumbL- or siControl-treated cells. (B) Quantification of Hes-1 mRNA levels in siNumb or siControl treated cells. Cells were treated with NG or GLT conditions for 12 h. *p<0.05 vs. siControl group under NG & ns; p>0.05. The data are representative from at least three independent experiments.
